# Amodiaquine-artesunate *vs *artemether-lumefantrine for uncomplicated malaria in Ghanaian children: a randomized efficacy and safety trial with one year follow-up

**DOI:** 10.1186/1475-2875-7-127

**Published:** 2008-07-11

**Authors:** George O Adjei, Jorgen AL Kurtzhals, Onike P Rodrigues, Michael Alifrangis, Lotte CG Hoegberg, Emmanuel D Kitcher, Ebenezer V Badoe, Roberta Lamptey, Bamenla Q Goka

**Affiliations:** 1 Department of Child Health, Korle Bu Teaching Hospital, University of Ghana Medical School, P. O. Box KB 4236, Accra, Ghana; 2 Polyclinic, Korle Bu Teaching Hospital, Accra, Ghana; 3 Ear, Nose and Throat Unit, Korle Bu Teaching Hospital, Accra, Ghana; 4 Centre for Medical Parasitology at Department of Clinical Microbiology and Department of Infectious Diseases, Copenhagen University Hospital (Rigshospitalet), and at Department of International Health, Immunology and Microbiology, University of Copenhagen, Denmark

## Abstract

**Background:**

Artesunate-amodiaquine (AS+AQ) and artemether-lumefantrine (AM-L) are efficacious artemisinin combination therapy (ACT) regimens that have been widely adopted in sub-Saharan Africa. However, there is little information on the efficacy of these regimens on subsequent episodes beyond 28 days, or on the safety of repeated treatments.

**Methods:**

Children aged six months to 14 years with uncomplicated malaria were randomly assigned to treatment with AS+AQ (n = 116), or AM-L (n = 111). Recruited subjects were followed-up, initially for 28 days, and then monthly for up to one year. All subsequent attacks of uncomplicated malaria after 28 days were treated with the same regimen as at randomization. Investigations aimed at determining efficacy and side effects were conducted.

**Results:**

Adequate clinical and parasitological response in subjects with evaluable end-points were, 97.1% (100/103) and 98.2% (107/109) on day 14, and 94.2% (97/103) and 95.3% (102/107) on day 28 in the AM-L and AS+AQ groups, respectively. Similar results were obtained after PCR correction. The incidence of malaria attacks in the year following recruitment was similar between the two treatment groups (p = 0.93). There was a high incidence of potentially AQ-resistant parasites in the study area. The incidence of adverse events, such as pruritus, fatigue and neutropaenia were similar in the two treatment groups. No patient showed signs of hearing impairment, and no abnormal neurological signs were observed during one year of follow-up. Other adverse events were mild in intensity and overlapped with known malaria symptomatology. No adverse event exacerbation was observed in any of the subjects who received multiple treatment courses with these ACT regimens during one year follow-up.

**Conclusion:**

AS+AQ and AM-L were efficacious for treatment of children with uncomplicated malaria in Ghana and drug-related adverse events were rare in treated subjects during one year of follow-up. The high prevalence of potentially AQ resistant parasites raises questions about the utility of AQ as a partner drug for ACT in Ghana. The efficacy of AS+AQ in Ghana requires, therefore, continuous monitoring and evaluation.

**Trial registration:**

NCT 00406146

## Background

Artemisinin-based combination therapy (ACT) may slow the development of resistance and reduce malaria transmission [1]. Artesunate-amodiaquine (AS+AQ) and artemether-lumefantrine (AM-L) are two ACT regimens that have been widely adopted for the treatment of uncomplicated malaria across sub-Saharan Africa. The efficacies of these two regimens are well established in trials of 14 or 28-day duration [2-4].

The efficacy of an ACT may depend on the level of pre-existing resistance to the non-artemisinin component [5], and it has been shown that, addition of an artemisinin derivative to a failing antimalarial drug leads to an ineffective combination [6]. AS+AQ, which is the ACT that has been adopted for first-line treatment of uncomplicated malaria in Ghana [7], is considered efficacious when the level of pre-existing resistance to AQ does not exceed 20% [8]. However, there is declining efficacy of AQ monotherapy in Ghana, with a recent in vivo efficacy study of AQ monotherapy in Ghana showing a cure rate below 50% [9].

The molecular basis underlying the mechanism of AQ resistance has not been fully elucidated, but *in vitro *and *in vivo *evidence suggests that AQ resistance is linked to the *T76 *single nucleotide polymorphism in the *Plasmodium falciparum *chloroquine resistance transporter (*Pfcrt*) [10,11]. Recently, a high *Pfcrt T76 *prevalence has been reported from Ghana [12], raising questions about the suitability and regional life span of AQ as a component of ACT in Ghana. There is however only one published report on the efficacy of AS+AQ from Ghana to date [13].

In spite of accumulating clinical experience and increasing use of ACT, aspects of the safety of these regimens remain a concern. With respect to artemisinins, high dose parenteral administration of the oil-soluble derivatives is associated with dose-dependent toxicity to areas of the brain stem involved in coordination and balance in animals [14,15]. No convincing evidence of artemisinin neurotoxicity has been demonstrated during routine clinical use in humans. However, reports of abnormal neurological signs in individual cases – each of which had underlying pre-morbidity, or received concomitant therapy, have been ascribed to artemisinin-based treatment in adults [16-19]. There is lack of longitudinally collected data on neurological findings in artemisinin-treated subjects, especially in children, the sub-group of malaria-treated patients that could be more susceptible to potential neurotoxic injury [20].

With respect to AQ, prophylactic use of this drug has been associated with fatal cases of agranulocytosis and hepatitis, mainly in non-immune adults [21,22]. Although it is considered that agranulocytosis and hepatitis are unlikely to occur when AQ is used for treatment [23], neutropaenia [24], as well as hepatitis [25], have been reported after administration of treatment doses of AQ, alone or in combination with AS. There is a lack of data on the natural history and clinical significance of reduced neutrophil counts during AQ treatment, and there is no data on possible cumulative neutrophil toxicity after repeated administration of treatment doses of AQ.

This study, conducted to generate efficacy and safety data on AS+AQ and AM-L to support the new ACT policy in Ghana, was a clinical trial with extended follow-up [26]. The design of the study allowed potential late-onset side effects, as well as the effects of repeated treatments on potential side effect occurrence to be evaluated. In addition, polymerase chain reaction (PCR) genotyping was done to determine baseline haplotypic frequencies of *Pfcrt *alleles in the study population.

## Methods

### Study site

The study was conducted in two primary health facilities, Korle Bu Polyclinic (KBP) and Mamprobi Polyclinic (MP). KBP is attached to a tertiary referral hospital, Korle Bu Teaching Hospital (KBTH). The two facilities are situated in Ablekuma sub-district in Accra, Ghana. Both facilities serve a mixed, predominantly low socio-economic urban population. The study was conducted between October 2004 and December 2006.

### Study population

Children presenting with a history of fever to the facilities were referred to the study team. A project physician examined the child to exclude concomitant illnesses. Subjects were recruited if a blood film was positive for *Plasmodium falciparum*, and if the study criteria were fulfilled.

### Criteria

Inclusion criteria for the study were, age six months to 14 years; signs and symptoms of acute uncomplicated malaria, including axillary temperature ≥37.5°C; confirmed *P. falciparum *mono-infection with parasite density between 2,000 – 200,000/μL; and willingness to comply with the follow-up schedule. Exclusion criteria were, symptoms or signs of severe malaria; obvious clinical evidence of chronic malnutrition or other severe disease; known intolerance or allergy to study medications; and reported treatment with any of the drugs under study one month preceding enrolment. Children aged five to 14 years were included to permit objective neurologic examination and audiologic assessment. Schoolchildren aged five to 14 years from two local primary schools were enrolled as controls for the audiometric study described below. Written informed consent was obtained from parents or guardians of all subjects. Ethical approval for the study was granted by the Ethical and Protocol Review Committee of the University of Ghana Medical School.

### Randomisation and blinding

A computer-generated simple randomization scheme was prepared in advance. Allocated treatments were kept in sealed opaque envelopes. After completion of formal enrolment procedures, allocated treatments were administered by project nurses. All study personnel (except project nurses), were unaware of the assigned treatments.

### Treatment allocation

AQ (Camoquine^®^, Pfizer, Dakar, Senegal), 10 mg/kg body weight, single daily dose for three days, was administered with AS (Plasmotrim^®^, Mepha Ltd, Aesch-Basel, Switzerland), 4 mg/kg body weight, single daily dose, for three days. AM-L (Coartem^®^, Novartis Pharma AG, Basel, Switzerland; 20 mg artemether and 120 mg lumefantrine) was administered at 0 and 8 hours on the first day, and then twice daily for two subsequent days according to body weight: 5–14 kg, one tablet/dose; 15–24 kg, two tablets/dose; 25–34 kg, three tablets/dose; 35 kg and over, four tablets/dose.

Project nurses administered all treatments (except the evening dose of Coartem) in the clinic. Parents administered the evening dose of Coartem^® ^at home [27,28]. Children were observed for 30 minutes after each drug administration. Treatments were re-administered if the child vomited. Children who vomited the re-ad ministered dose were withdrawn. An extra Coartem^® ^dose was given to parents to re-administer at home if the child should vomit.

### Follow-up

Subjects were followed-up on days 1, 2, 3, 7, 14 and 28 for clinical and laboratory assessments described below. Children who missed their day 14 or day 28 follow-up appointments were seen on days 15 or 29. After 28 days, children were visited at home every month for up to one year. During the monthly follow-up visits, parents or caregivers were asked about the child's well-being, behavioural and developmental concerns, as well as open questions about possible side event occurrence since the last visit. A study physician examined children during the follow-up visits. Follow-up was terminated if parents relocated. Each time a subject presented with an acute febrile illness during follow-up, thick films for malaria parasitaemia and a thorough examination was done, before and after the episode. All episodes of uncomplicated malaria diagnosed more than 28 days after treatment were treated with the same pre-assigned regimen as at randomization.

### Investigations

#### Neurological examination

Neurological examinations were done before treatment, and then on days 1, 2, 3, 7, 14 and 28. The neurological examination was focused on detecting abnormalities in areas that have been implicated in artemisinin toxicity in animals, i.e. on coordination and balance. In assessment for nystagmus, the target was always kept within binocular vision; the direction of the fast phase of nystagmoid eye movements was recorded; and it was noted if nystagmus was reduced by fixation or not. Any associated characteristics, such as vertigo, nausea, vomiting, and tinnitus were noted and recorded. Gait analysis, Romberg's test, speech assessment and the finger-nose test were done to evaluate cerebellar function. The finger-nose test was done for children aged six years or older. The responses for the finger-nose test were scored numerically for, 1) quality, i.e. smoothness and signs of intention tremor during the movement, and ii) adequacy, i.e. the successful implementation of the given task. The scores assigned for smoothness were, 0 = no tremor; 1 = slight tremor towards the end of the movement; and 2 = marked tremor increasing towards the end of the movement. The scores ascribed for adequacy were, 0 = the subject puts fingertip/pen top correctly at tip of nose each time; 1 = subject misses the tip of his/her nose/pen top once or twice; 2 = subject misses the tip of his/her nose/pen top each time. A score of zero for smoothness and adequacy was considered optimal for children aged six years and above, and all tests were scored with reference to the child's developmental age [29].

#### Audiometry

Pure tone, air conduction audiometry was done with a portable screening audiometer (Kamplex KS8, P.C. Werth, London, UK) for children aged five years and above who could cooperate with the test, on days 0, 3, 7, and 28. Hearing assessment was repeated after a follow-up period ranging from nine months to one year. Hearing thresholds were determined for each ear at the following frequencies: 125 Hz, 250 Hz, 500 Hz, 750 Hz, 1000 Hz, 1500 Hz, 2 KHz, 3 KHz, 4 KHz, 6 KHz and 8 KHz.

#### Laboratory methods

Venous blood was collected into EDTA and heparinized tubes on days 0, 3, 7, 14, 28 and on any day of recurrent symptomatic parasitaemia. Parasite counts were determined in Giemsa-stained blood films relative to 200 white blood cells (WBC) and the measured WBC count. Total WBC and differential counts were measured by automated haematology analyzer (Cell Dyn, Abbott Laboratories, USA). Serum aminotransferase and total bilirubin were measured by a clinical chemistry analyzer (EOS Bravo plus, Hospitex diagnostics, Italy).

#### Parasite genotyping

A nested PCR method was used to analyse polymorphisms in the merozoite surface protein (MSP) genes, *msp-1 *and *msp-2*, to distinguish between recrudescent and new infections, as described previously [30]. Nested PCR with sequence specific oligonucleotide probe (SSOP) ELISA was used to detect single nucleotide polymorphisms in the "C 72–76" position of the *Pfcrt *gene, as described previously [31].

### Efficacy outcome

The primary end-point was the unadjusted and PCR-adjusted cure rates at days 14 and 28. Efficacy outcomes were classified as, adequate clinical and parasitological response (ACPR), early treatment failure (ETF), late clinical failure (LCF) and late parasitological failure (LPF) [32].

### Secondary endpoints

Secondary endpoints were proportion of subjects with fever and parasitaemia 24 and 48 hours after treatment, the total number of subsequent malaria attacks in each arm, and incidence of adverse events before and after 28 days.

All adverse events were graded by severity (mild, moderate, severe, life-threatening), and relationship to study medication (unrelated, unlikely, possible, probable, definitely related) [33].

### Statistical analyses

The sample size was estimated on the assumption of a negligible (3%) risk of treatment failure with AM-L (because AM-L was new in the study area), and a 15% risk of AS+AQ treatment failure (because of prior extensive use of AQ monotherapy in the area). A sample size of 103 subjects was required to detect a 12% difference in cure rates between AS+AQ and AM-L with 95% confidence and 80% power. Categorical variables were compared with the chi square test with Yates correction or the Fisher's exact test. Continuous variables were compared with the student's t test or one-way analysis of variance. A logistic modelling (GENMOD™) procedure was used to compare the total number of treatments in the two arms for the year of follow-up. Risk differences and 95% confidence intervals were calculated with CIA™. Other analyses were done with Sigma stat (version 3.1; Systat software Inc., Richmond, California, USA) and SAS (SAS Institute, Cary NC, USA).

The primary efficacy outcome was analysed by a per-protocol (PP), and an intention-to-treat (ITT) method. In the ITT analysis, subjects lost to follow-up were included; these, as well as those withdrawn because of non-tolerance of oral medication, were considered treatment failures. In the PP analysis, only subjects with an assigned outcome were included. The follow-up time of subjects who did not complete one year of follow-up were censored at their last follow-up date. P values < 0.05 were considered significant.

## Results

### Base line characteristics

**Figure 1:**
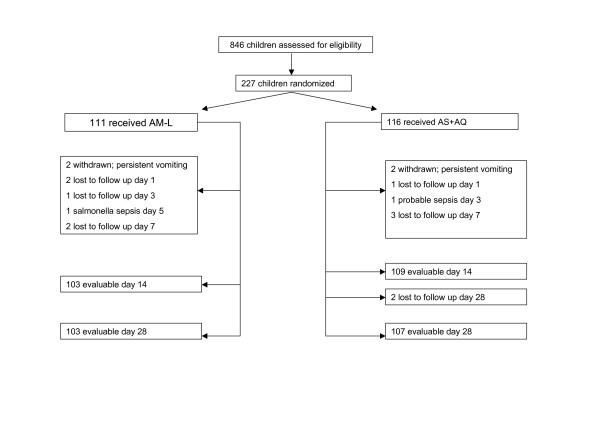
**Trial profile**. AM-L = artemether-lumefantrine, AS+AQ = artesunate+amodiaquine.

A total of 227 subjects were randomized out of 846 subjects screened. Sixteen subjects who were correctly randomized did not complete 28 days follow-up. The reasons for non-completion of follow-up in the AM-L arm were, withdrawal due to persistent vomiting necessitating parenteral treatment (n = 2); culture positive co-infection with *Salmonella typhi *resulting in worsening clinical condition after parasite clearance (n = 1); and loss to follow-up (n = 5). The reasons for non-completion of follow-up in the AS+AQ arm were, withdrawals due to persistent vomiting (n = 2); withdrawal due to worsening clinical condition despite rapid parasite clearance in an infant who was subsequently admitted and managed clinically for suspected septicaemia (n = 1); and loss to follow-up (n = 6). The flow of subjects through the study (up to day 28) is outlined (Figure 1). There were no differences in demographic and clinical parameters between the two groups (Table 1). The proportion of subjects between the ages of five and 14 years was 50.4% (56/111) in the AM-L arm and 55.2% (64/116) in the AS+AQ arm (p = 0.56).

**Table 1:** Baseline characteristics of recruited subjects

Characteristic	AM-L n = 111	AS+AQ n = 116	p
Male (n)	59	60	
Female (n)	52	56	
Age* (years)	5.2 (3–9)	6 (3.5–9.1)	0.72
Weight# (Kg)	19 (14–27.75)	18 (15–26)	0.66
Temperature# (°C)	38.6 (37.92–39)	38.7 (37.80–39.55)	0.19
Haemoglobin* (g/dL)	11.38 (2.21)	11.18 (1.96)	0.51
Parasitaemia# (/μL)	51408 (14377–115979)	60648 (20704–116496)	0.73
Total WBC count # (/μL)	8.9 (6.3–11.85)	9.1 (6.5–12.4)	0.95
Absolute neutrophil count# (/μL)	5411 (3541–7810)	5809 (4012–8236)	0.59

* Standard deviation, # range

### Treatment outcomes

#### Day 14 and 28 cure rates

The proportion of subjects (PP analysis) with ACPR were, 97.1% (100/103) and 98.2% (107/109) on day 14 (p = 0.67); and 94.2% (97/103) and 95.3% (102/107) on day 28 (p = 0.94) in the AM-L and AS+AQ groups, respectively (Table 2). The corresponding ACPR rates after PCR correction (PP analysis) on day 28 were, 96.1% (99/103) and 98.1% (105/107) (p = 0.43), respectively. The PCR-corrected cure rates by ITT analysis on day 28 were, 90.1% (100/111) and 91.3% (106/116) in the AM-L and AS+AQ groups (0.91), respectively. There were no differences in cure rates between younger children and children older than five years.

**Table 2:** Efficacy outcomes (per protocol analysis) on days 14 and 28

Day 14	AM-L	AS+AQ
ACPR	100	107
ETF	0	0
LCF	1	0
LPF	2	2

Day 28		

ACPR	97	102
ETF	0	0
LCF	3	1
LPF	3	4

AM-L = artemether-lumefantrine, AS+AQ = artesunate+amodiaquine

Day 28 cure rates are cumulative figures from day 14. ACPR = adequate clinical and parasitological response, ETF = early treatment failure, LCF = late clinical failure, LPF = late parasitological failure

#### Parasite clearance

**Figure 2:**
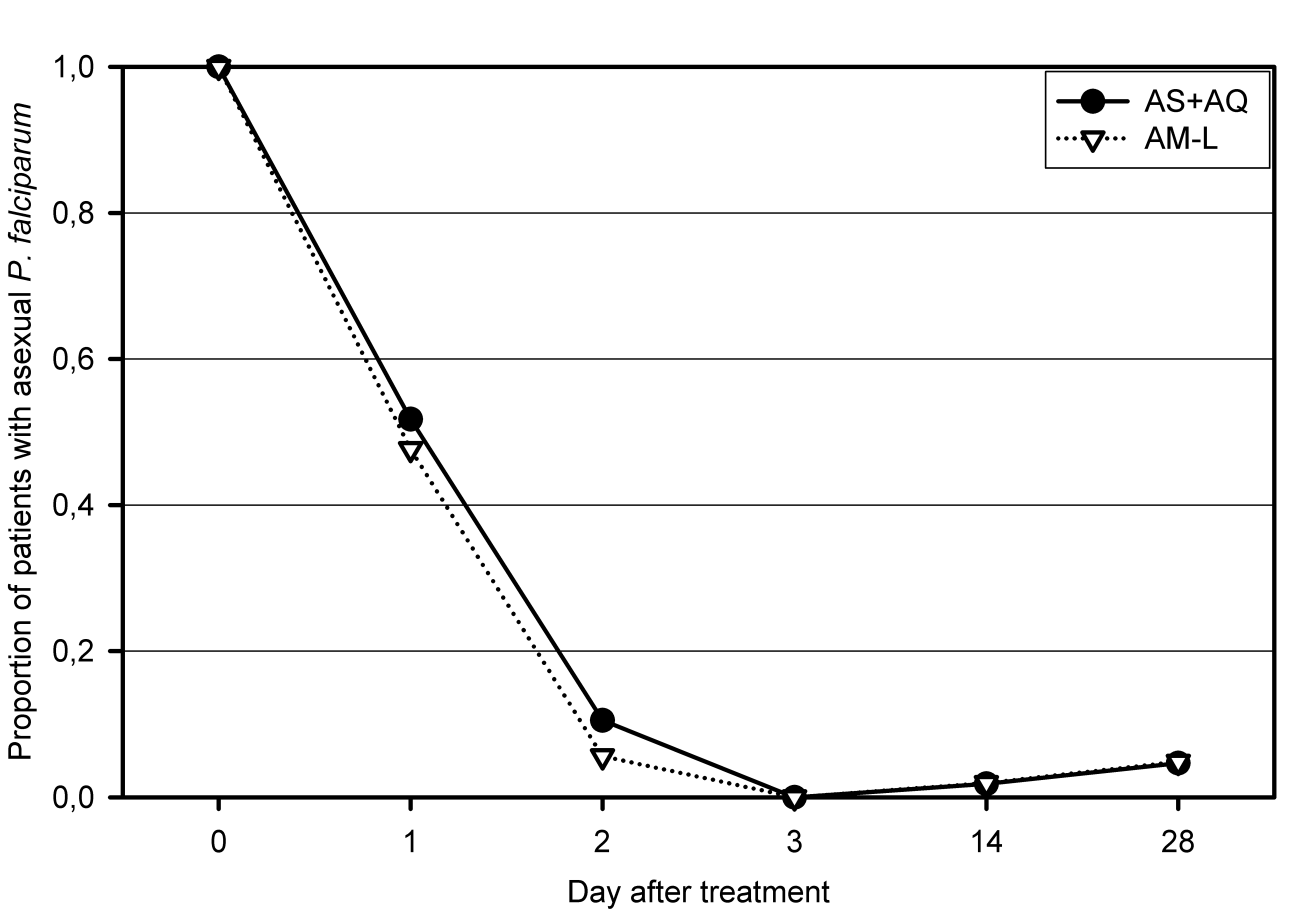
**Parasite clearance in the two groups**. AS+AQ = amodiaquine+artesunate, AM-L = artemether lumefantrine.

Parasite clearance on days 1 and 2 (Figure 2) were similar in the two groups.

#### Fever clearance

The proportion of subjects with fever was lower (though not significantly) in the AS+AQ group after 24 hours (5.3%) than in the AM-L (14.6%) group (p = 0.06). The trend had disappeared after 48 hours (p = 0.32).

### Symptomatic infections after day 28

After one year of follow-up, there were 34 further episodes of symptomatic uncomplicated malaria in 93 subjects (incidence rate, 0.37 per year) in the AS+AQ group, and 28 episodes in 82 subjects (incidence rate, 0.34 per year) in the AM-L group (incidence ratio, 0.93). Similar results were obtained, when logistic modelling (p = 0.94), or survival analysis (p = 0.76) was used to compare incidence in the two arms. All subjects who received repeat treatment courses responded appropriately to treatment. The time to a repeat treatment episode (median; interquartile range) was longer in the AS+AQ (91 days; 53–193 days) arm than the AM-L (64 days; 33–88 days) arm. There were three sibling pairs among those who were treated for repeat infections. These siblings (each randomized to receive a different ACT) reported simultaneously with a repeat attack of uncomplicated malaria.

### Pfcrt haplotypes

The "CVIET" (mutant) haplotype was present in 72.1% (75/104) of pre-treatment samples in subjects treated with AS+AQ. The effect of the "CVIET" haplotype prevalence on treatment outcome could not be evaluated because of the low number of subjects with treatment failure.

### Safety assessment

#### Audiometry

Audiometry could be done in 72 subjects (AS+AQ, n = 37; AM-L, n = 35). Hearing thresholds were significantly elevated in treated subjects than in age- and sex-matched control children on days 0, 3, 7, and 28, but there were no differences between hearing thresholds of subjects and controls after nine to 12 months. The full details of the audiometric study will be presented in another report.

### Neurological examination

Neurological examination could be done for 168 children (AS+AQ, 92; AM-L, 76). Nystagmus was observed in two subjects (AM-L, n = 1, AS+AQ, n = 1) between days one and seven. A detailed history indicated that one of these two subjects (7-year-old male; AS+AQ treated) was admitted as a neonate into neonatal intensive care (NICU) because of probable birth asphyxia. This subject was still attendant at pre-school at seven years of age (which is unusual in Ghana), and demonstrated excessive emotional lability. An electroencephalography (EEG) done for this subject because of a history suggesting the possibility of a seizure disorder, reported the presence of "epileptiform loci and generalized cerebral dysfunction". The other subject with nystagmus (9-year-old male, AM-L treated), also had a history of NICU admission for macrosomia (birth weight 4.5 kg) and possible neonatal hypoglycaemia. This subject had poor academic performance (had repeated first and second grades in school) and possible cognitive impairment. A routine EEG done for this subject was normal. A positive Romberg's test (which may suggest sensory ataxia) was noticed on day 3 in an 11-year old female (AM-L). This child was not attending school (despite her age) and had a history suggestive of possible cognitive impairment. No other abnormal neurological findings were observed in the remaining children during the 28-days follow-up, monthly follow-up visits, or for subjects who received multiple treatments.

#### Neutrophil counts

**Figure 3:**
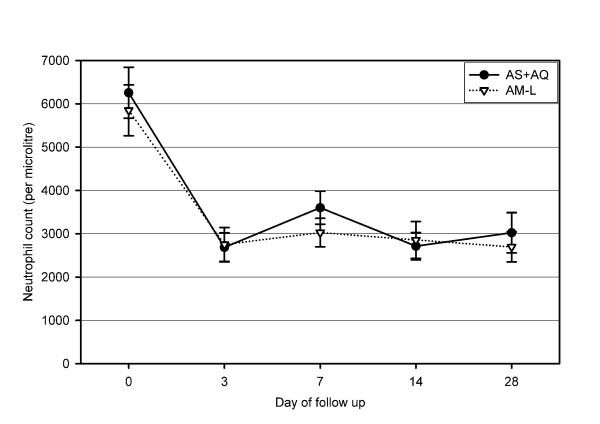
**Neutrophil count profiles in the two groups**. AS+AQ = amodiaquine+artesunate, AM-L = artemether-lumefantrine. Vertical error bars represent 95% confidence intervals.

**Figure 4:**
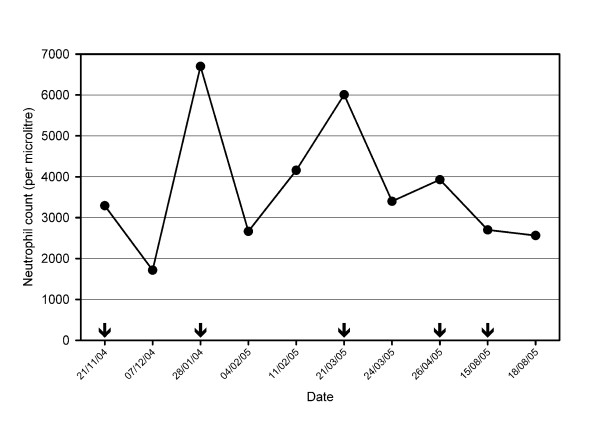
**Neutrophil count profile in a subject who was treated for five episodes of uncomplicated malaria with AS+AQ**. arrows point to the dates the subject presented with episodes.

There were no differences in the mean total WBC or absolute neutrophil counts between the two groups on days 0, 3, 7, 14 or 28 (Figure 3). Neutrophil counts on follow-up days 3, 7, 14 and 28 were significantly lower (p < 0.01) compared with day 0 counts in both treatment arms. Pre-treatment neutropaenia was observed in three subjects (AM-L, n = 2; AS+AQ, n = 1) on admission. Between days 3 and 28, neutropaenia was observed in 13 (11.7%) and 14 (12.6%) subjects in the AS+AQ and AM-L groups, respectively (p = 0.90). The median pre-treatment neutrophil count of subjects who developed neutropaenia was lower than subjects who did not develop neutropaenia, but the mean age was similar. Neutropaenia was severe (<500/μL) in three subjects (AM-L, n = 2; AS+AQ, n = 1) between days 3 and 28. Neutrophil counts did not show any pattern of difference between the initial and subsequent episodes of uncomplicated malaria in subjects who were treated more than once (data not shown). In two subjects, each of which was treated for five episodes of uncomplicated malaria (with AS+AQ) during the one-year follow-up, no clinical signs suggestive of neutropaenia was noticed, and all neutrophil count measurements were higher than 1,000/μL in these two subjects. The neutrophil count profile of one of these individuals is shown (Figure 4).

### Clinical chemistry

Serum alanine aminotransferase, aspartate aminotransferase and total bilirubin were initially (day 0) elevated, but there were no differences between the two treatment groups. The liver enzymes did not increase in response to treatment, but showed a trend towards normalization (reduction) as clinical symptoms improved (data not shown).

### Other adverse events

Within the first three days of treatment, complaints of dizziness were reported by 14 (12%) and eight (7.2%) subjects in the AS+AQ and AM-L groups, respectively (p = 0.31). Complaints of fatigue and excessive sleepiness were reported by five (4.3%) and four (3.6%) subjects in the AS+AQ and AM-L groups, respectively (p = 1.0). Pruritus was reported by three (AS+AQ, n = 2; AM-L, n = 1) subjects. A history of vomiting and nausea was part of the presenting complaint by 44.8% (52/116) and 40.5% (45/111) subjects respectively in the AS+AQ and AM-L groups on admission (p = 0.74). Between the first three days of treatment, 27.5% (32/116) and 12.6% (14/111) subjects respectively complained of vomiting and or nausea in the AS+AQ and AM-L groups (p = 0.01). Severe anaemia (haemoglobin 4.8 g/dl) occurred on day 14 in a 1.5 year old subject (AS+AQ). Severe anaemia and dizziness were considered unlikely to be drug related, but pruritus and excessive sleepiness were considered possibly drug-related. Other reported adverse events were mild in intensity, and overlapped with known malarial symptomatology. These were mostly classified as unrelated to study medications.

## Discussion

This study provides efficacy data on AS+AQ and AM-L, the two most widely adopted ACT regimens in sub-Saharan Africa, for Ghanaian children. The study also provides longitudinally collected safety data on neutrophil counts and neurological findings for Ghanaian children with uncomplicated malaria treated with repeated courses of these two ACT regimens.

The results showed a high cure rate for both regimens after a standard 14- or 28-day follow-up. These results are consistent with efficacy results reported from several sub-Saharan African countries [2-4,34], indicating a high overall cure rate with these regimens in the short (28-day) term. Because efficacy estimates could vary depending on the statistical analyses used to assess treatment outcome [35], and because recent guidelines recommend a change in treatment policy when failure rates exceed 10% [8], the use of the PP as well as an ITT analysis provided efficacy estimates that could be considered robust and operationally useful.

The finding of a high prevalence of potentially AQ-resistant parasites in this southern Ghanaian population raises questions about the long-term utility of AQ as a partner drug for ACT in Ghana, a question that has also been raised by other investigators from northern Ghana [9], where a similarly high *Pfcrt *mutant allele prevalence has been detected [12]. Although the impact of mutant *Pfcrt *allele prevalence on treatment outcome in the group treated with AS+AQ could not be assessed because of the low incidence of treatment failures in this group, AS+AQ has been associated with a high cure rate in an area of even higher (90%) *Pfcrt K76T *mutant prevalence [36]. This suggests that the high cure rate of the AS+AQ combination is due mainly to the effect of the artemisinin component. Recent evidence also suggests a differential selective pattern of parasite sensitivity for different ACT regimens [37]. Thus, it may be difficult to predict, or extrapolate *in vivo *efficacy of AS+AQ from one area to another, highlighting the need to generate local efficacy data for these newly introduced treatment regimens in Ghana. Furthermore, the high mutant *Pfcrt *allele prevalence also provides additional evidence justifying the treatment policy change in Ghana to ACT.

Previous studies that have compared AS+AQ with AM-L over a follow-up duration of 28-days or longer have demonstrated a lower re-infection rate for AM-L [2,4]. Other studies that have compared AM-L with ACT regimens consisting of longer-acting partner drugs have demonstrated a shorter time to re-infection for AM-L [38-40]. The longer time to a repeat clinical episode in the AS+AQ arm in this study could reflect the differences in chemoprophylaxis afforded by AQ, which has a relatively longer half-life. The possible implication of this finding is that, follow-up times required to detect subtle differences in efficacy between different ACT regimens, may need to be lengthened.

There was a drop in neutrophil counts in both arms after treatment. This pattern of neutrophil count reduction, as well as the absence of differences in neutrophil count profiles between children who received single course and those who received multiple courses, suggests the possibility of a disease-rather than drug-related effect.

Malaria-associated neutropaenia has been reported for subjects treated with AQ, either as monotherapy, or in combination with AS [24], as well as for subjects treated with AM-L [41] or with other antimalarials [42]. Neutrophil count reduction during acute malaria may be due to altered intravascular granulocyte distribution [43], or to enhanced phagocytosis [44]. AQ-associated agranulocytosis on the other hand, may be due to inhibition of bone marrow precursor cells [45,46], or to immune-mediated hypersensitivity [47,48]. The finding that neutrophil counts were not increasingly reduced in subjects who received more than one treatment course in this study lends support to the conclusion that, brief three-day administration of AQ, as used for treatment, is unlikely to be associated with the described side effects observed during prophylaxis [23]. However, there was a large random variability around the confidence interval of the difference in neutropaenia incidence between the groups, and the sample size of the study was not sufficient to detect rare events; therefore, this potential side effect should be continuously monitored.

This is one of the few studies to conduct systematic longitudinal neurological examinations for children treated with artemisinin-based regimens. A total follow-up lasting 12 months did not reveal abnormal neurological signs that could be ascribed to artemisinin-based treatment in this study. The two subjects with observed nystagmus had underlying conditions that could easily explain the noted abnormalities. Furthermore, the observed nystagmus was not associated with other cerebellar signs, suggesting these were unlikely to be of clinical significance, or could be false positive due, for instance, to excessive lateral eye abduction. However, nystagmus has been reported in artemisinin-treated subjects without reported underlying neurological abnormality [49], as well as in an experimental study of artemisinin-treated rhesus monkeys [50]. Because available data from animal studies do not provide much insight into the early signs of possible artemisinin-related neurotoxicity, and because of the lack of a well-defined neurological syndrome to characterize potential late artemisinin toxicity during clinical use, detailed focused studies on this subject are still required.

The incidence of gastrointestinal adverse events was higher in the AS+AQ group compared to the AM-L group, similar to a recent report comparing AS+AQ with dihydroartemisinin-piperaquine [51]. This may suggest a lower intrinsic tolerance of AS+AQ, or could be due to the differences in drug formulation, as AS+AQ was a co-administered regimen, whilst the AM-L was co-formulated.

The limitations of this study include its limited statistical power to detect rare side effects, as well as the difficulties associated with evaluating neurological function in the younger children below five years of age. Further studies of the nystagmoid eye movements, apart from the clinical observations done, would also be useful.

## Conclusion

AS+AQ and AM-L were efficacious for the treatment of Ghanaian children with uncomplicated malaria. After 12 months of detailed systematic follow-up, no overt clinical evidence of neurotoxicity or neutropaenia was observed. However, the sample size was limited for conclusive evidence on safety; therefore, post-marketing surveillance, with particular emphasis on neurological and haematological changes, is still indicated. In view of the high prevalence of potentially AQ-resistant mutant parasites in the study area, the efficacy of the AS+AQ combination, the first-line treatment for uncomplicated malaria in Ghana, requires continuous monitoring and evaluation.

## Authors' contributions

GOA, BQG, OPR and JALK designed the study; GOA, BQG, OPR, EVB, EDK and RL did the clinical work. GOA, LCGH and MA did the laboratory analyses. GOA and JALK did the data analysis. GOA drafted the manuscript, and all authors contributed significantly to the final draft.
